# When to Choose In Vitro Fertilization (IVF) When Postponing Conceiving: The Age‐Related Risk of IVF Failure at an Advanced Age

**DOI:** 10.1155/ogi/2407539

**Published:** 2025-12-15

**Authors:** Zhiyan Chen, Duoduo Zhang, Zhengyi Sun, Qi Yu, Chenyang Zhao, ChangZhong Li

**Affiliations:** ^1^ Department of Obstetrics & Gynecology, Peking University Shenzhen Hospital, Shenzhen, China, pkuszh.com; ^2^ Department of Obstetrics & Gynecology, Chinese Academy of Medical Sciences and Peking Union Medical College Hospital, Chinese Academy of Medical Sciences and Peking Union Medical College, Shenzhen, China, cacms.ac.cn; ^3^ Medical Technology Department, The Islands Healthcare Complex - Macao Medical Center of Peking Union Medical College Hospital, Macao, China; ^4^ Institute of Obstetrics and Gynecology, Shenzhen PKU-HKUST Medical Center, Shenzhen, China, pku.edu.cn; ^5^ Shenzhen Key Laboratory on Technology for Early Diagnosis of Major Gynecologic Diseases, Shenzhen, China

**Keywords:** age, fertility, in vitro fertilization (IVF)

## Abstract

**Background:**

To validate previous age subgrouping methods and provide an optimal age reference for women planning to delay in vitro fertilization (IVF).

**Methods:**

From July 2014 to March 2018, 3012 patients that received IVF/intracytoplasmic sperm injection (ICSI) were continuously recruited in this retrospective, single‐center study. We analyzed the relationships of baseline characteristics and IVF outcomes. A smooth fitting curve depicting the association of age and live birth was plotted using the generalized additive model (GAM) method. We also evaluated the association of age and live birth among different age groups (> 20, ≤ 35; > 35, ≤ 37; > 37, ≤ 40; and > 40).

**Results:**

Age, duration of infertility, and baseline follicle‐stimulating hormone (FSH) were significantly related to the live birth rate. The adjusted Odd Ratio (OR) value of age was 0.95 (*P* < 0.001), indicating the higher occurrence of no live birth with age increasing. The fitting curve showed that the live birth rate decreased with age. And 35.5 (34.5–36.5) was identified as the inflection point of the curve. The slopes before 35.5 and after 35.5 were significantly different (0.9 [0.9, 1.0], *P* < 0.001). When ≤ 35, live birth rate did not vary with age. For the ages of 35–37 and 37–40, the occurrence of negative outcome increased with age (aOR: 0.73 [0.53, 0.99], 0.80 [0.65, 1.00]; *P* = 0.0441, 0.0465, respectively).

**Conclusion:**

The age of 35 can be referred to as the safe time point when the IVF success rate does not decline with age. And the risk of IVF failure increases rapidly in the period of 35–37 and 37–40.

## 1. Background

Infertility has become a worldwide health problem, which constitutes the primary cause of population decline in many countries [1]. Female age has been broadly recognized as the main factor that affects the success rate of spontaneous conception [2]. However, with the accelerating development of modern society, recent years have witnessed an increasingly popularized tendency of postponing childbearing. A large number of women have chosen higher education or profession in their 20s or 30s instead of motherhood [3]. As a consequence, more females begin to resort to assisted reproductive technologies (ARTs), such as in vitro fertilization (IVF)/intracytoplasmic sperm injection (ICSI), to address their aging‐related infertility. Unfortunately, the effectiveness of those medical methods is often overestimated [4]. The truth is that the overall reproduction success rate for both spontaneous fertility and assisted reproduction is highly dependent on maternal age [5]. Adverse pregnancy outcomes, including miscarriage and chromosomal abnormalities, are positively associated with increasing age. The aneuploid blastocysts, mainly caused by advanced maternal age (AMA), can directly increase implantation failure and other delivery risks after IVF [6, 7]. The weakened viability of oocytes also precipitates the bad IVF outcomes of older women [8]. AMA, defined as the age above 35 years old, is widely used as the turning point to mark the upsurging risk of infertility. And previous studies have also reported that it could be referred to as the guidance for predicting the success rate of IVF [9–13].

Confronting with the dilemma of postponing conceiving of women and higher occurrence rate of IVF failure in AMA, a more detailed proposal to guide the infertile women who have IVF plans can be of significant clinical value. Therefore, we launched this study exploring the specific relationship between age and IVF outcomes based on a Chinese medical center’s data. We divided the recruited patients into different age groups to examine the changes in successful IVF outcomes in this group according to previous reports [14, 15]. We also evaluated other possible factors at baseline that might be potentially relevant to the outcomes. We hope that on the basis of the study, we can provide evidence‐based guidance about the optimal reference age for IVF and the corresponding estimated chance for fertility and delivery when counseling the patients.

## 2. Methods

### 2.1. Participant Recruitment

The study was designed as a retrospective, single‐center one, and it was approved by the Institutional Review Board of Peking Medical College Hospital (No. S‐K601). Written informed consent was signed by each recruited patient.

From July 2014 to March 2018, we continuously recruited patients who received IVF/ICSI from the Assisted Reproduction Clinic, Peking Medical College Hospital. The patients were diagnosed with infertility of tubal disorders or male causes. The exclusion criteria are the patients with polycystic ovarian syndrome, endometriosis, previous endocrine disorders, such as pituitary tumors, thyroid abnormalities, and patients with malignancy history and mental illness. The patients with PCOS and endometriosis were excluded to minimize potential confounding effects, as both conditions are known to significantly and independently influence IVF outcomes through distinct mechanisms.

For those recruited patients, a gonadotropin‐releasing hormone agonist (GnRHa) long protocol was enacted to suppress ovarian function and synchronize the menstrual cycle, making it easier to control and predict the timing of ovulation. During the IVF process, eggs were retrieved from the ovaries and fertilized with sperm in a laboratory setting. The acquired embryos are then allowed to develop for a few days before being transferred back into the uterus. For the patients receiving ICSI, a single sperm was directly injected into an egg to improve the chances of fertilization, particularly in cases of male infertility.

All of the embryos acquired from the IVF/ICSI cycles were transferred before the study was conducted.

### 2.2. Baseline Clinical Characteristics and Confirmation of the Fertility Outcomes

Baseline clinical characteristics of the patients, including age, body mass index (BMI), and infertility duration, were recorded. Serum sexual hormonal levels at baseline of the treatment process, including human follicle‐stimulating hormone (FSH), estrogen (E2), luteinizing hormone (LH), prolactin (PRL), and testosterone (T), were tested on the 2nd day of the menstrual cycle using radioimmunoassays.

The variables for pregnancy outcome included number of oocytes retrieved, number of mature oocytes, number of two‐pronuclear zygotes, number of cleavage‐stage embryos, number of top‐quality embryos on the 3rd day, number of blastocyst‐stage embryos, clinical pregnancy, and live birth. Clinical pregnancy was defined as ultrasonic validation of the existence of gestational sac and fetal heartbeats. Live birth was defined as live births greater than 28 gestational weeks. The determination of clinical pregnancy and live birth was based on the cumulated outcome of all stimulating cycles.

### 2.3. Statistics

R (https://www.R-project.org) and EmpowerStats software (X&Y Solutions) programs were applied to perform the statistical analysis. The mean ± standard deviation (SD) was used to describe continuous variables, and percentages (%) were used to express categorical variables. *p* values < 0.05 were considered statistically significant.

To observe the change of live birth with age, spline smoothing based on the generalized additive model (GAM) was utilized to illustrate the two variables’ relationship graphically and capture potential nonlinear patterns. The GAM curve was adjusted for other relevant variables to control for confounding effects, ensuring that the observed relationship is more accurately attributed to age. A maximum likelihood estimate (MLE) was used to calculate the injection point of the smoothing curve. This point is significant as it indicates where the nature of the relationship between age and live birth rate changes. To enhance the robustness of the estimates, a bootstrap resampling method that randomly selected 500 samples from the dataset was performed to obtain 95% confidence intervals (CIs) along with MLE. We then divided the curve into two segments by the inflection point, showing the difference of the relationship between age and live birth before and after this critical age threshold. The Wald test was used to examine the null hypothesis that the coefficients of the segments are equal to zero, which would indicate no relationship between age and live birth rate in those intervals.

Based on previous studies showing that 37 years and 40 years were also crucial points of alteration of pregnancy outcomes [14, 15], we divided the patients into four age groups, listed as follows: > 20, ≤ 35; > 35, ≤ 37; > 37, ≤ 40; and > 40. To compare the clinical characteristics at baseline, we utilized Student’s *t*‐test for comparing the continuous variables, the Kruskal–Wallis test for the continuous variables among multiple groups, and Fisher’s exact test for categorical variables. The univariate analysis was firstly carried out to examine the possible factors correlated with the live birth rate. Then we selected the generalized estimating equation (GEE) model to explore the associations between the characteristics and outcomes. This approach is suitable given the dataset’s structure, which included patients undergoing multiple cycles. The results of the GEE analysis were presented as adjusted odds ratios (aORs) and 95% CIs for assessing the associations between the clinical characteristics and live birth outcomes.

## 3. Results

We recruited a total of 3012 patients with a mean age of 34.9 ± 4.3 who underwent IVF/ICSI in this single‐center retrospective study, among which 2101 received IVF cycles and 911 received ICSI. The clinical characteristics at baseline before treatment are presented in Table 1. There are a total of 1631 women who had live birth and 1381 with no live birth among recruited patients. From the univariate analysis result in Table 1, we found that age and baseline FSH are statistically related to pregnancy outcomes. The live birth group and the no live birth group showed no significant differences in other factors, including BMI, duration of infertility, baseline T, E2, PRL, LH, and types of ART method.

**Table 1:** Baseline clinical characteristics of the recruited patients.

	Live birth	No live birth	*p* value
Number	1631	1381	
Age	35.32 (4.46)	34.43 (4.13)	< 0.001
BMI	22.03 (3.20)	22.16 (3.11)	0.123
Duration of infertility	4.00 (0.50–24.00)	4.00 (0.50–21.00)	0.256
T	0.41 (0.06–20.10)	0.40 (0.00–10.98)	0.744
E2	45.78 (4.50–442.00)	46.28 (5.23–591.90)	0.357
PRL	16.15 (0.47–152.00)	16.00 (0.54–76.05)	0.782
LH	3.76 (0.20–60.56)	3.71 (0.20–32.84)	0.504
FSH	8.13 (3.92)	7.60 (3.33)	< 0.001
Method			0.732
IVF	1142 (70.02%)	959 (69.44%)	
ICSI	489 (29.98%)	422 (30.56%)	

*Note:* FSH: human follicle‐stimulating hormone, E2: estrogen, PRL: prolactin, T: testosterone, and ICSI: intracytoplasmic sperm injection.

Abbreviations: BMI = body mass index, IVF = in vitro fertilization, LH = luteinizing hormone.

We examined the association of each clinical factor and the outcomes after adjusting other factors, presented as aOR in Table 2. Age, duration of infertility, and baseline FSH were significantly related to the live birth rate (aOR of age: 0.95 [0.94, 0.97], *p* < 0.0001; aOR of duration of infertility: 0.97 [0.95, 0.99]. *p* = 0.00119; aOR of FSH: 0.96 [0.94, 0.98], *p* = 0.0001). The aOR value of age was 0.95, indicating a higher occurrence of no live birth with age increasing. Table 3 demonstrates the associations of age with other pregnancy outcome indicators. Age was significantly negatively correlated with all of the indicators of fertility outcomes (aOR < 1, *p*  <  0.0001).

**Table 2:** The association of each clinical factor and the outcomes after adjusting.

	Statistics	aOR	*p* value
Age (years)	34.91 ± 4.33	0.95 (0.94, 0.97)	< 0.0001
BMI (kg/m^2^)	22.09 ± 3.15	1.01 (0.99, 1.04)	0.2525
Duration of infertility (years)	4.72 ± 3.07	0.97 (0.95, 0.99)	0.0119
Method			
IVF	2101 (69.75%)	1.0	
ICSI	911 (30.25%)	1.03 (0.88, 1.20)	0.7317
FSH (IU/L)	7.89 ± 3.67	0.96 (0.94, 0.98)	0.0001
T (ng/mL)	0.47 ± 0.72	0.91 (0.80, 1.03)	0.1462
E2 (pg/mL)	51.02 ± 32.36	1.00 (1.00, 1.00)	0.4051
PRL (ng/mL)	17.92 ± 9.16	1.00 (0.99, 1.01)	0.5742
LH (IU/L)	4.29 ± 3.09	0.98 (0.96, 1.01)	0.1571

*Note:* FSH: human follicle‐stimulating hormone, E2: estrogen, PRL: prolactin, T: testosterone, and ICSI: intracytoplasmic sperm injection.

Abbreviations: aOR = adjusted odds ratio, BMI = body mass index, IVF = in vitro fertilization, LH = luteinizing hormone.

**Table 3:** The associations of age with the pregnancy outcome indicators.

	aOR (age and outcomes)	*p* value
No. of oocytes retrieved	−0.39 (−0.43, −0.35)	< 0.0001
No. of mature oocytes	−0.34 (−0.38, −0.31)	< 0.0001
No. of two‐pronuclear zygotes	−0.31 (−0.35, −0.27)	< 0.0001
No. of cleavage‐stage embryos	−0.31 (−0.34, −0.27)	< 0.0001
No. of top‐quality embryos on the 3rd day	−0.04 (−0.05, −0.03)	< 0.0001
No. of blastocyst‐stage embryos	−0.15 (−0.17, −0.13)	< 0.0001
Clinical pregnancy	0.96 (0.94, 0.97)	< 0.0001
Live birth	0.96 (0.94, 0.97)	< 0.0001

Abbreviation: aOR = adjusted odds ratio.

**Figure 1:**
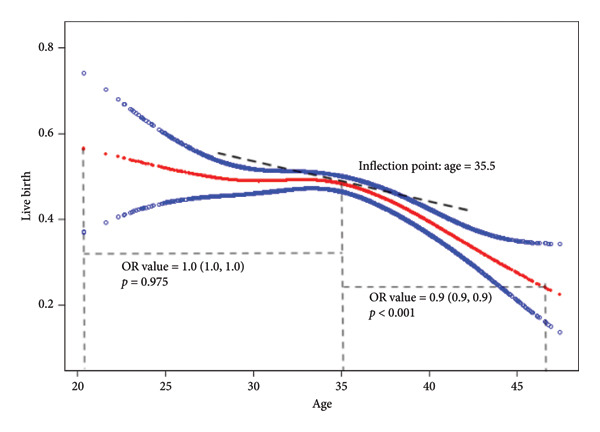
The smooth curve depicting the relationship of live birth and age. *X*‐axis: age; *Y*‐axis: live birth probability. The live birth probability decreased with age. And the curve was flatter before a certain point. The inflection point is age of 35.5. A more rapidly declining slope after a plateau period was shown. The OR value of age is 1.0 (1.0, 1.0) before the inflection point, with a *p* value of 0.975. The OR value of age is 0.9 (0.9, 0.9) after the inflection point, with a *p* value < 0.001.

We plotted a smoothing curve to depict the relation of age and live birth rate in Figure 1. Overall, the live birth probability decreased with age. However, we found that although the smoothing curve showed an overall declining trend, it flattened out before a certain point. And there exhibited a more rapidly declining slope after a plateau period. The age of 35.5 (95% CI: 34.5–36.5) was calculated to be the injection point of the curve. The OR value of the curve slope before 35.5 was 1.0 (1.0, 1.0) (*p* = 0.975), and the OR value of the curve slope after 35.5 was 0.9 (0.9, 0.9) (*p*  <  0.001). To further emphasize the significance of this finding, the slopes before and after 35.5 years were compared. The slopes before 35.5 and after 35.5 were significantly different (0.9 [0.9, 1.0], *p*  <  0.001), highlighting the steeper decline in live birth rate after reaching this critical age.

Subsequently, we stratified the patients into four age groups, listed as follows: > 20, ≤ 35; > 35, ≤ 37; > 37, ≤ 40; and > 40. The associations between age and live birth rate of the four age groups are presented in Table 4. For the group of ≤ 35, the live birth rate did not vary with age, indicating that age had a negligible impact on the fertility outcomes within this group. For the group of 35–37 and 37–40, the occurrence of negative outcome built up with aging (aOR: 0.73 [0.53, 0.99], 0.80 [0.65, 1.00; *p* = 0.0441, 0.0465, respectively). This suggests that for those recruited women, the risk of live birth decreased by 27% and 20% per year in the ages of 35–37 and 37–40, respectively. For those patients over 40 years, the live birth rate reached a relatively low level. Within this cohort, no statistically significant association between age and live birth rate was detected.

**Table 4:** The associations between age and live birth rate of the four age groups.

Age groups	Number of live birth	Live birth
< 35	1567 (52.03%)	1.01 (0.97, 1.05) 0.7059
≥ 35, ≤ 37	554 (18.39%)	0.73 (0.53, 0.99) 0.0441
> 37, ≤ 40	487 (16.17%)	0.80 (0.65, 1.00) 0.0465
> 40	404 (13.41%)	0.89 (0.77, 1.02) 0.0996

## 4. Discussion

In this study, on the basis of the observational data of IVF/ICSI‐receiving women from a Chinese medical center, we performed a systematic analysis about the association between age and IVF outcomes, and we also scrutinized other possible factors that might have an impact on the outcomes from the baseline clinical characteristics. A fitting curve displaying the relationship of age and IVF/ICSI outcomes was plotted using the method of spline smoothing.

The age of 35.5 was identified as the curve inflection point of the GAM plot reflecting the relationship of age and live birth. For the ages of 35–37 and 37–40, the risk of IVF failure increased significantly with age. And after 40 years, no significant association was detected between age and live birth. The above results can be referred to when selecting the optimal age for IVF.

A remarkable transit in the demographics of maternal women is distinguished globally in these years [16]. According to a survey in Canada, the age group with the highest fertility rate was 30–35 after 21 centuries, which was < 30 in the 90s. And the number of women that gave their first birth at the age of 35–40 increased from 4% to 11% [17]. Although more modern women are choosing to delay the age of childbearing, they do not fully understand the risks associated with having children at an advanced age [18]. Not only natural conception but also the success rate of ART is also strongly influenced by age. And for those who reach the age of 45, a positive outcome of IVF rarely occurs [19]. It has been well established that 35 years old, defined as AMA, is an important point to assess female fertility for both spontaneous pregnancy and ART, which is anticipated to undergo an age‐related decline after the point. Hence, intense evaluation and medical intervention are recommended for AMA women with the intention of conceiving. It has also been estimated that downfall in fecundity can also be observed after the age of 37 [14]. One study about IVF‐receiving patients in the US found that the proportion of live births was 41.5% among women under 35, 31.9% among women aged 35–37, 22.1% among women aged 38–40, and 18.4% among women over 40 [20]. Given the previous results, we speculate that the ages of 37 and 40 are also critical time points for fertility assessment.

To confirm the findings of previous literature and further explore the clinical values, we performed this observational, retrospective study and divided the patients into four age groups for comprehensive analysis. Firstly, we validated that 35.5 is the turning point of the changes in age‐related IVF outcomes. In the 35–37 and 37–40 age groups, the live birth rate will decline rapidly with age. Reaching a certain low value after the age of 40, the decline rate will gradually become slower. Based on our findings regarding the age‐related risks associated with IVF/ICSI outcomes, we could propose several specific recommendations for patient consultation. The women aged > 35 years could face a higher risk of lower live birth rates compared to younger cohorts. Gynecologists could help the patients make informed decisions about the time of IVF/ICSI, especially for those who are not ready to receive ART but wish to in the future. And those patients should also be aware that there is a significant decrease in success rates between the ages of 35–37 and 37–40. This time period should be taken into consideration when planning IVF.

There are several limitations in this study. Firstly, selection bias existed in this study, as it was designed as a retrospective study. And the amounts of recruited patients and cycles were relatively small in this single‐center study. A rigorously designed prospective cohort study with sensitivity analysis should be performed in future research to further validate the conclusion in this study. Also, the exclusion of patients with PCOS and endometriosis may limit the generalizability of our results. Moreover, in this study, the clinical variables for the baseline were relatively simple and insufficient. For instance, the husband’s age and semen quality, smoking history, and sociological information were not collected. In addition, only FSH was used for the evaluation of the patient’s ovarian functional reserve, while AMH was not assessed. Another limitation of our study is the absence of detailed recording and analysis of other pregnancy‐related outcomes, such as ectopic pregnancy and miscarriage. In further study, more variables should be included, such as AMH levels, semen parameters, the age of the male partner, lifestyle factors, and uterine factors, to provide a more comprehensive evaluation of factors influencing IVF outcomes.

In conclusion, in this study, based on the investigation of the age‐related IVF outcomes in patients from a Chinese medical center, we proposed an age range that is optimal to receive IVF for Chinese women. After 35 years, the age‐related risk of IVF failure can be significant. And in the period of 35–37 and 37–40, live birth rate decreases by 27% and 20% per year. We also attempt to make the patients aware that aging is strongly associated with IVF failure and poor fertility outcomes, in addition to the impact on natural fertility.

**Nomenclature**
IVF: In vitro fertilization
ICSI: Intracytoplasmic sperm injection
GAM: Generalized additive model
FSH: Follicle‐stimulating hormone
OR: Odd Ratio
ART: Assisted reproductive technology
AMA: Advanced maternal age
GnRHa: Gonadotropin‐releasing hormone agonist
BMI: Body mass index
E2: Estradiol
COH: Controlled ovarian hyperstimulation
hCG: Human chorionic gonadotropin
LH: Luteinizing hormone
PRL: Prolactin
T: Testosterone
CVs: Coefficients of variation
SD: Standard deviation
MLE: Maximum likelihood estimate
CIs: Confidence intervals
GEE: Generalized estimating equation

## Ethics Statement

The Institutional Review Board of Peking Medical College Hospital approved the retrospective observational study (No. S‐K601). Written informed consent was obtained from the patients.

## Disclosure

All authors have read and approved the final manuscript.

## Conflicts of Interest

The authors declare no conflicts of interest.

## Author Contributions

DZ collected and validated the patient data. ZC analyzed and interpreted the patient data and was a major contributor in writing the manuscript. ZS and YQ supervised the study. CZ and CL supervised the study and revised the manuscript.

## Funding

This study was supported by the Shenzhen High‐Level Hospital Construction Fund (YBH2019‐260), Shenzhen Key Medical Discipline Construction Fund (No. SZXK027), and Sanming Project of Medicine in Shenzhen (No. SZSM202011016).

## Data Availability

The datasets used and/or analyzed during the current study are available from the corresponding author on reasonable request.
